# Evaluation of an X-Ray Dose Profile Derived from an Optically Stimulated Luminescent Dosimeter during Computed Tomographic Fluoroscopy

**DOI:** 10.1371/journal.pone.0132154

**Published:** 2015-07-07

**Authors:** Hiroaki Hasegawa, Masanori Sato, Hiroshi Tanaka

**Affiliations:** 1 Department of Bioinformatics, Graduate School of Medical and Dental Sciences, Tokyo Medical and Dental University, Bunkyo-ku, Tokyo, Japan; 2 Department of Radiological Sciences, Graduate School of Health Sciences, Komazawa University, Setagaya-ku, Tokyo, Japan; 3 Department of Bioinformatics, Division of Medical Genomics, Medical Research Institute, Tokyo Medical and Dental University, Bunkyo-ku, Tokyo, Japan; Institute for Health & the Environment, UNITED STATES

## Abstract

The purpose of this study was to evaluate scatter radiation dose to the subject surface during X-ray computed tomography (CT) fluoroscopy using the integrated dose ratio (IDR) of an X-ray dose profile derived from an optically stimulated luminescent (OSL) dosimeter. We aimed to obtain quantitative evidence supporting the radiation protection methods used during previous CT fluoroscopy. A multislice CT scanner was used to perform this study. OSL dosimeters were placed on the top and the lateral side of the chest phantom so that the longitudinal direction of dosimeters was parallel to the orthogonal axis-to-slice plane for measurement of dose profiles in CT fluoroscopy. Measurement of fluoroscopic conditions was performed at 120 kVp and 80 kVp. Scatter radiation dose was evaluated by calculating the integrated dose determined by OSL dosimetry. The overall percent difference of the integrated doses between OSL dosimeters and ionization chamber was 5.92%. The ratio of the integrated dose of a 100-mm length area to its tails (−50 to −6 mm, 50 to 6 mm) was the lowest on the lateral side at 80 kVp and the highest on the top at 120 kVp. The IDRs for different measurement positions were larger at 120 kVp than at 80 kVp. Similarly, the IDRs for the tube voltage between the primary X-ray beam and scatter radiation was larger on the lateral side than on the top of the phantom. IDR evaluation suggested that the scatter radiation dose has a high dependence on the position and a low dependence on tube voltage relative to the primary X-ray beam for constant dose rate fluoroscopic conditions. These results provided quantitative evidence supporting the radiation protection methods used during CT fluoroscopy in previous studies.

## Introduction

Because of increasing use of X-ray computed tomography (CT), the percentage of the total radiation dose to patients has become larger for CT than for other X-ray imaging procedures [1]. With respect to CT procedures, the X-ray exposure time in CT fluoroscopy has become a major concern [2, 3] because the examination time is longer for CT-guided interventional radiology (e.g., percutaneous needle biopsy and drainage) than for other CT diagnostic imaging procedures [4–6]. CT fluoroscopic images are displayed almost at the same time to enable real-time image reconstruction, which improves the accuracy of needle positioning in cases of large respiratory fluctuations that can affect the target lesion. In contrast with CT fluoroscopy, a conventional method uses a single-shot scan shows biopsy needle displacement caused by respiration (quick-check techniques [4]). On the other hand, personnel exposure cannot be avoided because the physician is required to operate devices by the side of the patient even during the CT fluoroscopic procedure. The personnel exposure is mainly caused by scatter of the primary X-ray beam irradiation intended for the patient.

To optimize personnel exposure protection, it is important to obtain information on detailed scatter radiation doses administered during CT fluoroscopy. However, in a previous study [7] on personnel exposure in CT fluoroscopy, scatter radiation was measured only indirectly as a function of the distance from the CT fluoroscopic section. To date, the dose relationship between the primary X-ray beam and scatter radiation associated with measurement on the subject surface has not been clarified. Measurements of detailed dose profiles of the CT fluoroscopic section in the direction toward the gantry is necessary. Dose profiles provide primary X-ray beam and scatter radiation dose distribution as a function of position, and enable measurement of the X-ray beam shape. Comparison of the integrated primary X-ray beam dose with scatter radiation using dose profile measurements enables accurate determination of the scatter radiation dose.

Measurement of dose profiles is performed using thermoluminescence dosimeters [8, 9] or Gafchromic film [10–13]. A dose profile is not measured using practical thermoluminescence dosimeters with high positional resolution. Although the film method is easier for measuring a dose profile, complex calibration procedures for dose estimation are necessary for accurate quantification [14, 15]. A pencil-type ionization chamber is used for measurement of integrated dose along the direction toward the gantry by only measuring air ionization within the collected volume. In contrast, optically stimulated luminescent (OSL) CT dosimeters have advantages for measurement of detailed dose profiles and are expected to be widely applied to radiation dosimetry in clinical sites [16–19]. The OSL CT dosimeter allows easy measurement of detailed dose profiles and is useful for dose estimation on the patient surface but was found in a previous study to be insufficient when performing ionization chamber dosimetry [20].

The irradiated detector is stimulated by green light (532 nm) and detects blue OSL emission (420 nm) from the Al_2_O_3_:C detector in the reader system. The conduction and valence bands are separated by a band gap in which only localized energy levels were introduced by defects in the crystalline structure. Because ionizing radiation yields electron–hole pairs, the radiation-induced electrons are free to move from the conduction band to the valence band and are captured by these energy levels in the crystal lattice. The trapped charges revert back to the conduction band by optical stimulation, which results in electron–hole pair recombination and luminescence. Thus, the stimulated luminescence intensity is a surrogate for the trapped charge concentration and, thereby, the absorbed dose [21]. The advantage of the OSL dosimeter is that it is easy to handle because OSL dosimeters do not require high-temperature heating, unlike thermoluminescence dosimeters [22].

The purpose of this study was to evaluate scatter radiation dose using an X-ray dose profile derived from OSL dosimetry measurements of the subject’s surface during CT fluoroscopy, a type of evaluation that has been difficult to perform previously.

## Materials and Methods

### OSL CT dosimeters

The OSL CT dosimeter used in this study had a long strip shape with a 150 mm (length) × 6 mm (width) × 0.35 mm (thickness) Al_2_O_3_:C detector (Landauer Inc., Glenwood, IL, USA). The holder had a diameter of 12 mm and a length of 175 mm. The measurement length was 150 mm, and the positional resolution was 0.05 mm. The detectable radiation range was ≤150 cGy.

### Phantom

CT fluoroscopy is often used in the thoracic region. A chest phantom (CTU-4, Kyoto Kagaku Co. Ltd, Kyoto, Japan) was used for the subject, and the soft tissue of the phantom was composed of polyurethane resin. The chest phantom was equipped with a simulated lung partitioned into right and left lobes and had a bone structure implanted on the back side to simulate the vertebrae. The chest phantom was placed on the couch so that the phantom center was 5 cm lower from the iso-center of the X-ray tube rotation. This means that the biopsy needle prevents collision with the gantry. This setting is usually used in CT-guided interventional radiology and is recommended for patient safety.

### Positioning for measurement

**Fig 1:**
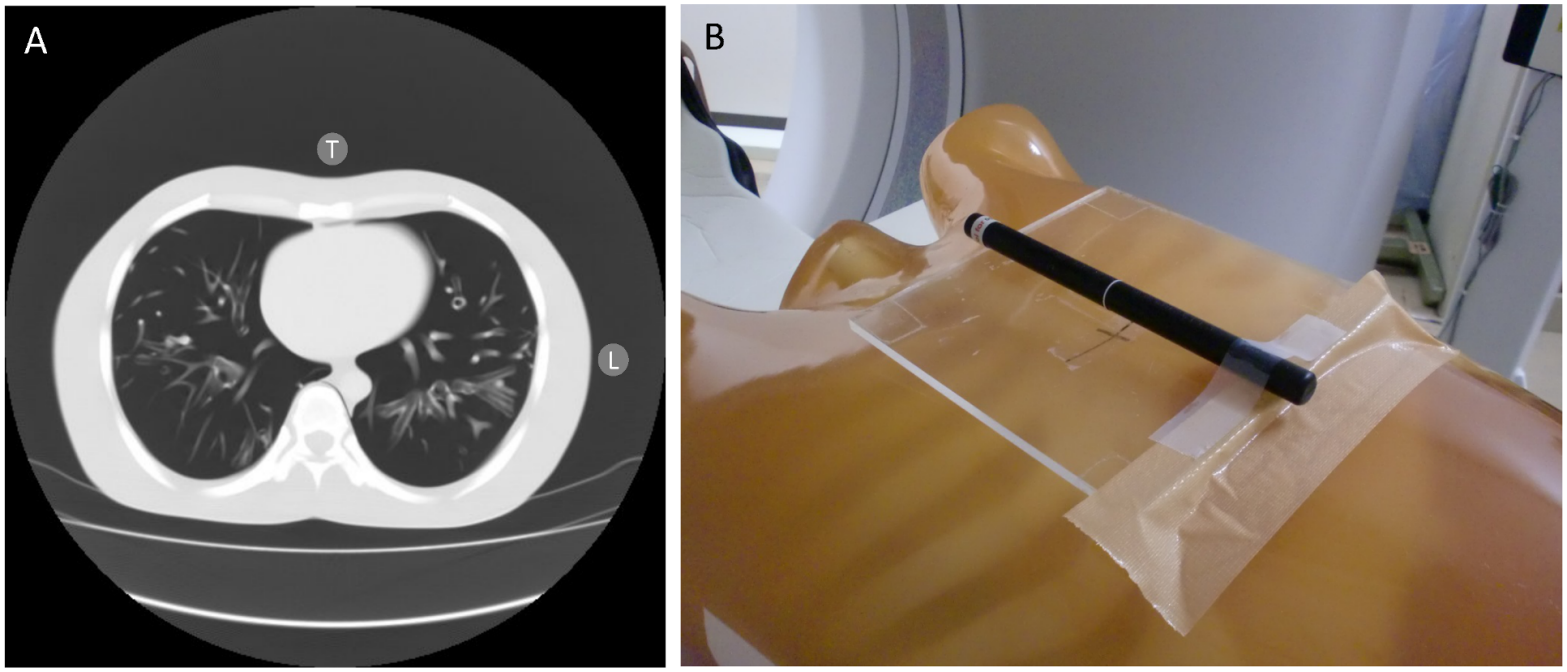
Measuring positions used for optically stimulated luminescence dosimeters. A: Computed tomography image of the chest phantom at the center position during fluoroscopy; T, the position of optically stimulated luminescence (OSL) dosimeters on the top of the chest phantom; L, the position of OSL dosimeters on the lateral side of the chest phantom. B: Photograph of a measurement on the top of the chest phantom.

OSL dosimeters were placed on the top and the lateral side of the chest phantom so that the center of measurement coincided with the X-ray beam, and the longitudinal direction of the OSL dosimeter was set parallel to the z-axis (orthogonal axis to the slice plane and directed towards the gantry). OSL dosimeters were placed on the top and the lateral side of the chest phantom, the strip surface was placed toward the center of the chest phantom (Fig 1).

### CT fluoroscopic conditions

This study was performed using a multislice CT scanner (Asteion 4, Toshiba Medical Systems, Otawara, Tochigi, Japan) in the CT fluoroscopic mode. We performed air calibration and confirmed that the CT unit could be used safely and properly before the study commenced. For clinical use, the constancy test (IEC361223-2-6) [23] was conducted regularly on the CT unit to assure the quality of measurements. The CT fluoroscopic mode was used for continuous X-rays in the preset position without moving the couch. CT fluoroscopy was performed under the following conditions: slice thickness, 12 mm (4 × 3-mm detector collimation); X-ray tube voltage, 120 kVp (thousands of volts peak) and 80 kVp; and tube rotation speed, 1 second. The CT fluoroscopic conditions were set to give a dose rate (mGy/sec) equal to that used as the reference displayed CT dose index (CTDI) [24] on the scanner. In relation to the collimated X-ray beam width, Boone [25] reported that using a Monte Carlo simulation in which 100-mm pencil chambers were capable of measuring the CTDI100, and until 40 mm X-ray beam width was acceptable. Thus, we used a 100-mm length integrated dose in our investigation. The CT fluoroscopic X-ray tube current was 40 mA (3.88 mGy/second) at 120 kVp and 110 mA (3.89 mGy/second) at 80 kVp. The CT fluoroscopic times were set to 15 seconds and 30 seconds for each tube voltage. Two different CT fluoroscopic times were set to observe the standard errors of the measurements. The absorbed dose is linearly proportional to the mAs (the product of the mA and exposure time in seconds). In this scanner, the X-ray beam shaping filter was different from the data collection field of view (CFOV) in the slice plane. In terms of patient exposure reduction, it is required to properly set the CFOV according to the patient size. Thus, because the major axis of the chest phantom slice plane was 28 cm in the center of the dose profile measurement, the CFOV was set to “medium” (maximum collection diameter, 40 cm). Boone also considered the blurring of dose distributions of the X-ray beam penumbra [25]. The penumbra depends on differences in the focus size and may affect the integrated dose of the scatter tail sections. We performed CT fluoroscopy using a small focal spot so as not to affect the X-ray penumbra results.

### Accuracy of integrated dose from OSL dose profiles

To verify the accuracy of OSL dosimetery, we compared the values of the integrated dose between OSL dosimetery and ionization chamber dosimetery (100 mm length; PTW, Freiburg, Germany) in each of the measurement positions. The dose profiles were obtained using the OSL dosimeter and not the ionization chamber. In this experiment, the measurements were made under eight conditions resulting from the combination of the following three parameters, each of which was varied between two settings: (1) measurement positions at the top and the lateral side of the chest phantom, (2) fluoroscopic tube voltages of 120 kVp and 80 kVp, and (3) fluoroscopy times of 15 seconds and 30 seconds. The total number of OSL dosimetry measurements was 24 because triplicate measurements were made for each condition, and the total number of measurements with the ionization chamber was 40 because five repeated measurements were made for each condition.

The dose profiles were calculated from the mean value of each OSL dosimetry measurement made at 0.05-mm intervals. Furthermore, the integrated dose was obtained from the average dose profiles of triplicate measurements. The integrated dose from the ionization chamber was calculated from the average of five repeated measurements. We determined the standard errors for the average of repeated measurements. The standard error was determined by the statistical analysis of a series of observations. The standard uncertainty was defined as the variation in the results of a measurement [26]. As reported by the manufacturer, the variation in the luminescence intensity of the OSL dosimeter was <5% between OSL elements that were irradiated with the same dose. In addition, when measuring the laser output variation, the fluctuation in the luminescence intensity, and the dark current noise during calibration, the expanded uncertainty (k = 2; k is the coverage factor) [26] when using OSL dosimeters was approximately 10%. Consequently, the uncertainties in the OSL measurements, including those of the X-ray generator output variation, and positioning errors were larger than the uncertainty in the OSL measurements alone. Moreover, the energy response changes caused the uncertainty to increase for lower tube voltages and scattering radiation because the OSL dosimeter was calibrated using a 120-kVp (aluminum half value layer: 8.3 mm) X-ray beam. In the measurements made using the ionization chamber, the sources of uncertainty were the reading of the dosimeter and quantity under measurement (e.g., air pressure and temperature, chamber-specific influence quantities, radiation field-specific influence quantities, and chamber positioning) [27]. These sources were also present in the cross calibration to determine the calibration coefficient of the ionization chamber. The expanded uncertainty (k = 2) when using the ionization chamber was approximately 10% for the calibration performed by our regional secondary standards dosimetry laboratories. In the CT dosimetry using the ionization chamber, the IAEA reports that the expanded uncertainty is as high as 14.4% when using the standard body (32-cm diameter) CT dosimetry phantom [28, 29]. In this instance, the uncertainty in the dosimeter’s intrinsic error was calculated to be 2.89% (k = 1) for cross calibration performed by the secondary standards dosimetry laboratories. We believe that the error estimate would be larger if the expanded uncertainties are included because (1) the intrinsic error of the ionization chamber was larger than that described in the previously mentioned IAEA’s literature and (2) the ionization chamber was positioned at the peripheral chest phantom. The variation in the X-ray output and the error in the dosimeter setup were considered to be the most significant factors in the measurement uncertainty when using any of the dosimeters. Hence, it was valid to assume that the uncertainties were the experimental standard error estimated on the basis of the repeated measurements, three OSL profiles, and five measurements of the ionization chamber.

D_OSL_ was defined as the integrated dose calculated from the dose profiles measured by OSL dosimeters, and D_IC_ was defined as the integrated dose calculated from the ionization chamber. The percent difference between the integrated doses calculated from OSL dosimeters and from the ionization chamber (ε) was calculated from the following equation:

$$\varepsilon \;=\;\frac{D_{OSL}\;-\;D_{IC}}{D_{IC}}\;\times \;100$$

The absolute value of ε was used for evaluation of the measurement errors. The OSL dosimetry measurement length was 150 mm, and the effective ionization length was 100 mm at the ionization chamber. The integration length was 100 mm, in compliance with CTDI_100_ [24]. D_OSL_ and D_IC_ were calculated from the cumulative integrated dose during the preset fluoroscopic time. Each OSL dosimeter was exposed and subsequently sent to the manufacturer (Landauer Inc.) for analysis. The manufacturer reported dose data as air kerma via electronic files. The pencil-type ionization chamber was calibrated using a 70-kVp (aluminum half value layer: 3 mm) X-ray beam from an industrial X-ray generator in the secondary standards dosimetry laboratories. A cross calibration using the pencil-type ionization chamber and a thimble-type ionization chamber (DC300; Wellhofer, Schwarzenbruck, Germany) for a secondary standard was performed, and the calibration coefficient was determined for the ionization chamber. Although the method of the pencil-type ionization chamber calibration has not yet been established, the calibration was performed using a lead sleeve equal to the ionization volume (0.3 cc) of the DC300 reference chamber. The calibration coefficient, including the air kerma conversion coefficient (8.404 × 107 Gy cm / C), was preset in the electrometer. The ionization charges were output from the electrometer. Therefore, the indicated value of the electrometer was the air kerma (Gy cm). The atmospheric air correction factor [23] (0.9937) was multiplied by the air kerma. The atmospheric correction factor was calculated from the atmospheric pressure and room temperature at the time of measurement. The ionization chamber was placed on the top and the lateral side of the chest phantom so that the X-ray beam was exposed to the center of the effective ionization length similar to the placement of OSL dosimeters.

### Dose evaluation of primary X-ray beam and scatter

In this study, to evaluate the scatter tail dose from the subject during CT fluoroscopy, the dose profiles were separately investigated by examining the integrated dose of the primary X-ray beam and the scatter radiation that was outside of the primary beam and that corresponded to the dose profile tails. Note that the scatter tail dose means the integrated dose for the scatter tail sections and does not include all the scattering from the phantom.

For an integrated dose and a 100-mm length, D_P_ was defined as the integrated dose of the primary X-ray beam width. D_S_ was defined as the integrated dose of the dose profile tails caused by scatter radiation distribution. D_P_ and D_S_ were calculated according to the following equations:

$$D_{P}\;=\;\int_{-\frac{\text{1}}{\text{2}}T}^{\frac{\text{1}}{\text{2}}T}\;D(z)\;dz$$

$$D_{S}\;=\;\;\int_{-50}^{-\frac{\text{1}}{\text{2}}T}\;D(z)\;dz\;+\;\int_{\frac{\text{1}}{2}T}^{50}\;D(z)\;dz$$

Accordingly, D_OSL_ was calculated according to the following equation:

$$D_{OSL}\;=\;\int_{-50}^{50}\;D(z)\;dz\;=\;D_{P}\;+\;D_{S}$$

where T is the nominal primary X-ray beam width and D(z) is the dose profile along the z-axis during the preset fluoroscopic time. Each IDR used for the scatter tail dose evaluation was calculated for each fluoroscopic time. IDR_S/T_ was defined as the ratio between D_OSL_ and D_S_. Similarly, IDR_P_ was defined as the ratio between the top and the lateral side of the chest phantom, and IDR_V_ was defined as the ratio between 120 kVp and 80 kVp. For D_P_ and D_S_, IDR_P_ was further decomposed into IDR_PP_ and IDR_PS_, and IDR_V_ was decomposed into IDR_VP_ and IDR_VS_, respectively.

### Statistical analyses

Statistical analyses were performed by two-way analysis of variance (ANOVA) for each fluoroscopic time for the integrated dose (D_P_ and D_S_). The fluoroscopic condition parameters (measurement positions and tube voltage) were regarded as independent variables. The significance of difference was analyzed for the main effects and for interaction effects. The level of statistical significance was set at 0.05. All statistical analyses were performed using JMP Pro 9.0.2. (SAS Institute Inc., Cary, NC, USA)

## Results

### Accuracy of integrated dose from OSL dose profiles

**Fig 2:**
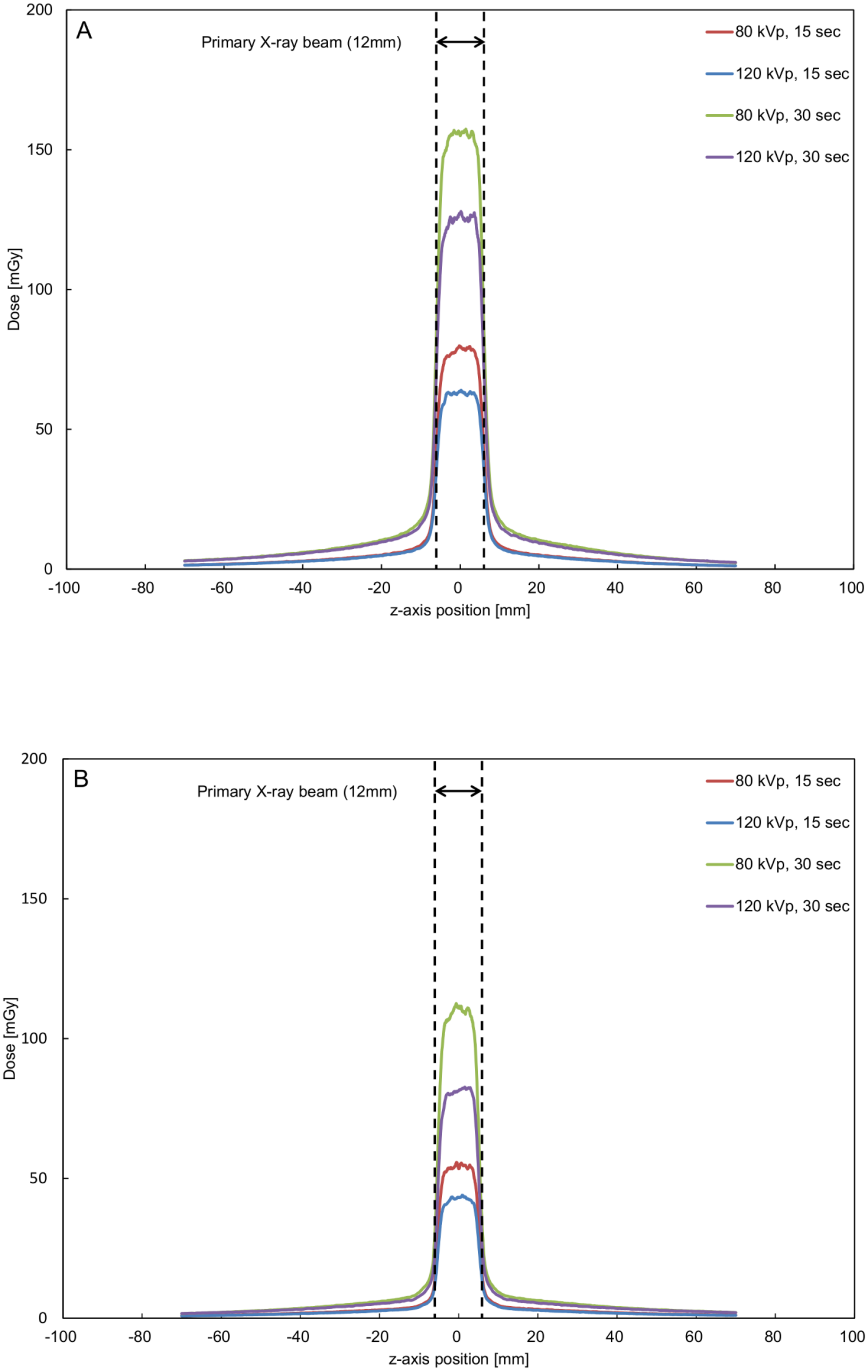
Dose profiles of CT fluoroscopy performed using optically stimulated luminescence dosimeters. A: Top. B: Lateral side. Each data point corresponds to the mean value of triplicate measurements.

The measured ionization chamber doses and OSL dosimetry doses for the eight fluoroscopic conditions are summarized in Table 1. The overall percent difference between the integrated doses from OSL dosimeters and from the ionization chamber (the absolute value of ε) was 5.92% (range, 0.01%–16.22%). The average percent difference was 5.51% on the top and 6.06% on the lateral side. These measurements were found to have a strong correlation (R^2^ = 0.9930, p < 0.0001). The OSL dosimetry dose profiles on the top and the lateral side of the phantom are shown in Fig 2.

**Table 1:** Integrated dose measurements performed using optically stimulated luminescence dosimeters and an ionization chamber.

Measurement conditions		Integrated dose^a^ (mGycm)				| ε |^d^ (%)	
		Ionization chamber^b^		OSL dosimeters^c^			
Tube voltage	Position	15 seconds	30 seconds	15 seconds	30 seconds	15 seconds	30 seconds
**80 kVp**	**Top**	134.04 ± 0.29	266.53 ± 0.36	134.06 ± 1.12	269.71 ± 5.78	0.01	1.19
	**Lateral side**	79.07 ± 021	158.72 ± 0.39	82.31 ± 0.61	165.88 ± 2.39	4.10	4.51
**120 kVp**	**Top**	127.68 ± 0.09	255.54 ± 0.08	113.59 ± 3.17	227.31 ± 3.00	11.03	11.05
	**Lateral side**	80.69 ± 0.37	160.51 ± 0.30	67.60 ± 1.11	130.18 ± 0.08	16.22	18.90

^a^Values are presented as the mean ± standard error.

^b^Mean value of five repeated measurements.

^c^Mean value of triplicate measurements.

^d^The absolute value of the percent difference was defined as (D_OSL_ − D_IC_)/D_IC_ × 100.

### Dose evaluation of the primary X-ray beam and scatter

The scatter tail doses from the phantom were evaluated under different conditions of measurement positions and tube voltage to determine the integrated dose and primary X-ray beam dose, and the IDRs for each condition were calculated.

**Fig 3:**
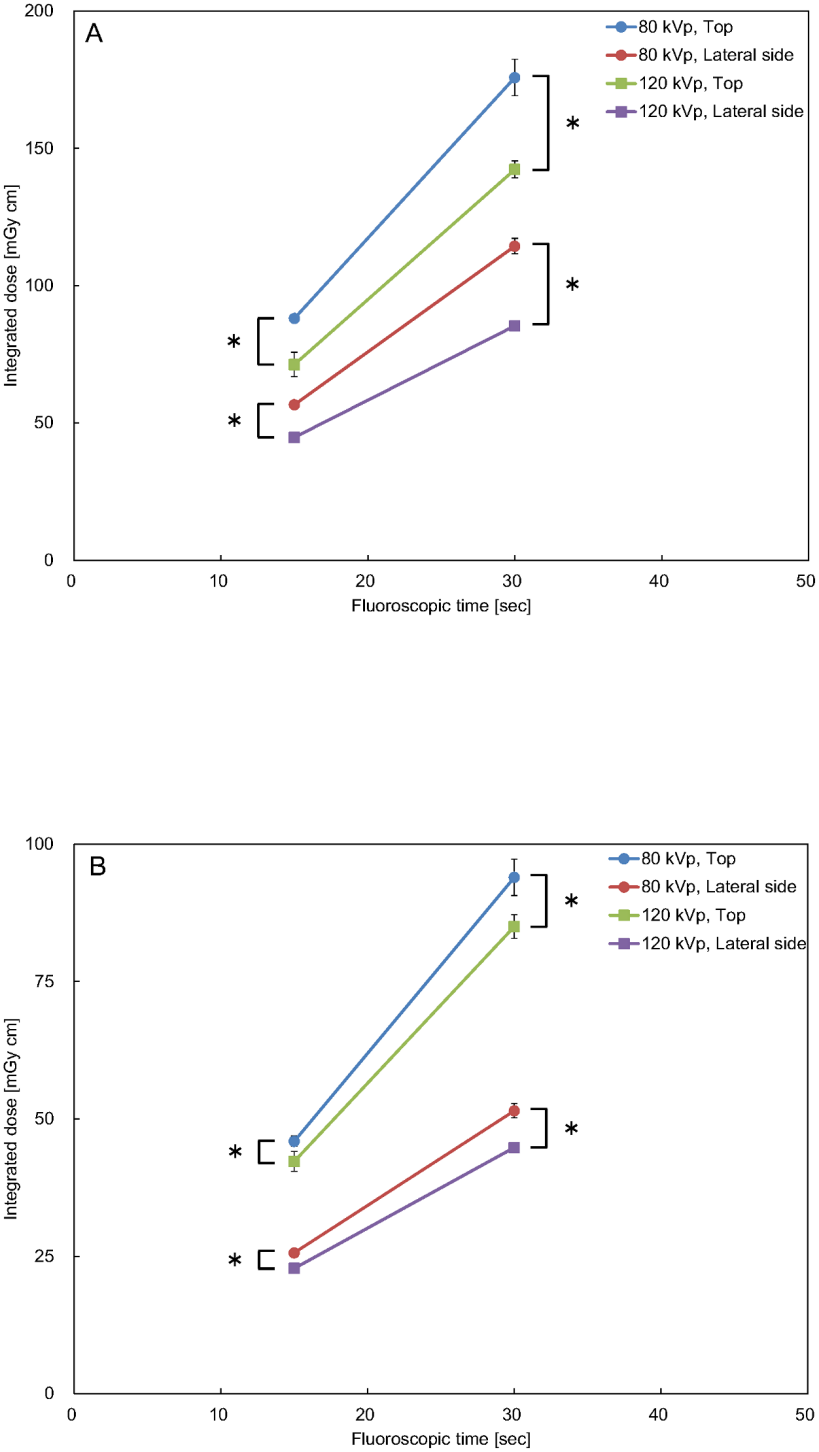
Integrated doses for the different measurement positions and tube voltage. There were statistically significant differences (* p < 0.0001) as determined by two-way ANOVA between the main effects (measurement positions and tube voltage) for each fluoroscopic time. Error bars depict standard error of triplicates. A: Primary X-ray beam. B: Scatter radiation.

The cumulative integrated doses for the measurement positions and tube voltage for the primary X-ray beam dose (D_P_) and the scatter tail dose (D_S_) are shown in Fig 3. The results of two-way ANOVA for each fluoroscopic time showed that there were significant differences in the fluoroscopic conditions between the main effect (measurement positions and tube voltage; p < 0.0001). The interactions did not show significant differences. IDR_S/T_ of the measurement positions and tube voltage are presented in Table 2. IDR_S/T_ was the lowest on the lateral side at 80 kVp and the highest on the top at 120 kVp. The IDR_P_ results are presented in Table 3, and the IDR_P_ was larger at 120 kVp than at 80 kVp. For the same fluoroscopic time, the IDR_PP_ and IDR_PS_ were 1.56 and 1.79 at 80 kVp and 1.59 and 1.85 at 120 kVp, respectively. The IDR_V_ results are presented in Table 4. The IDR_V_ was larger on the lateral side of the phantom than on the top. During the 15-second fluoroscopy, the IDR_VP_ and IDR_VS_ were 1.24 and 1.09 on the top of the phantom and 1.27 and 1.12 on the lateral side, respectively. A similar tendency was observed during the 30-second fluoroscopy.

**Table 2:** Integrated dose ratio (IDR_S/T_) of the 100-mm length integrated dose (D_OSL_) and scatter radiation dose (D_S_) determined by optically stimulated luminescence dosimetry.

Measurement conditions		IDR_S/T_ ^a^	
Tube voltage	Position	15 seconds	30 seconds
**80 kVp**	**Top**	0.34	0.35
	**Lateral side**	0.31	0.31
**120 kVp**	**Top**	0.37	0.37
	**Lateral side**	0.34	0.34

^a^The integrated dose ratio of the dose caused by scatter radiation distribution (outside the primary X-ray beam width) in the dose profile tails to that of the 100-mm length along the z-axis of the chest phantom was measured.

**Table 3:** Integrated dose ratio for the measurement positions (IDR_P_) with optically stimulated luminescence dosimetry.

Measurement conditions	IDR_PP_ ^a^		IDR_PS_ ^b^	
Tube voltage	15 seconds	30 seconds	15 seconds	30 seconds
**80 kVp**	1.56	1.54	1.79	1.82
**120 kVp**	1.59	1.67	1.85	1.90

The IDR_P_ is the ratio of integrated dose that measured each tube voltage on the top to that on the lateral side of the chest phantom.

^a^IDR_P_ in primary X-ray beam width along the z-axis at the chest phantom.

^b^IDR_P_ of the dose profile tails caused by scatter radiation distribution (outside of the primary X-ray beam width) along the z-axis of the chest phantom.

**Table 4:** Integrated dose ratio for the tube voltage (IDR_V_) determined by optically stimulated luminescence (OSL) dosimetry.

Measurement conditions	IDR_VP_ ^a^		IDR_VS_ ^b^	
Position	15 seconds	30 seconds	15 seconds	30 seconds
**Top**	1.24	1.23	1.09	1.11
**Lateral side**	1.27	1.34	1.12	1.15

The IDR_V_ is the ratio of the integrated dose measured at each position of the chest phantom at 80 kVp to that at 120 kVp.

^a^IDR_V_ of the primary X-ray beam width along the z-axis of the chest phantom.

^b^IDR_V_ of the dose profile tails caused by scatter radiation distribution (outside of the primary X-ray beam width) along the z-axis of the chest phantom.

## Discussion

Yukihara et al. [19] reported that the CTDI_100_ values based on the OSL dose profile agreed within ± 5% with the CTDI_100_ values based on a 100-mm long pencil ionization chamber. In the present study, the overall percent difference between the integrated doses from OSL dosimeters and from the ionization chamber agreed with that of a previous study although the measurement conditions were different. Furthermore, under a measurement condition used in clinical practice, the accuracy of the integrated dose from OSL dosimetry was equal to that from using an ionization chamber. Because there were no other dosimeters that can easily measure CT dose profiles with high resolution in the z-axis except for OSL dosimeters, further increased use of OSL dosimetry is expected.

We evaluated scatter radiation from the subject by calculating the IDR in the measurement positions and tube voltage.

The IDR_S/T_ indicated that the quantity of scattering was less at 80 kVp than at 120 kVp, and the value was less on the lateral side of the phantom than on the top. These results accord that the quantity of scattering depended on the tube voltage. The IDR_S/T_ was less on the lateral side of the phantom than on the top because of scatter absorption at the phantom. (The tube current was substantially different because the dose rates were equal between the fluoroscopic conditions. The quantity of scattering does not depend on the tube current although the scattering dose was increased at 80 kVp.) Therefore, to reduce exposures to personnel, the use of a low tube voltage that produced less scattering is recommended. Use of a lower tube voltage is a possible solution for substantial reduction of the radiation dose rate [30]; fluoroscopic conditions using lower tube voltages could yield personnel hand exposure reductions of ≥60%, as determined in a cylindrical acrylic phantom study [7]. In the present study, we used a phantom that resembled anatomical elements, and the lower voltage condition was effective in personnel exposure reduction.

The IDR_P_ value was larger at 120 kVp than at 80 kVp because scatter absorption at the phantom and scatter radiation. The IDR_PS_ value was larger than the IDR_PP_ value, which was caused by the difference in X-ray absorption that in turn depended on the X-ray beam travel length between the measurement positions because the photon energy of the scatter radiation was lower than that of the primary X-ray beam. Therefore, it appeared that the positional dependence for measurement of scatter radiation was larger than that for the primary X-ray beam. The positional dependence for measurement of scatter radiation was larger at 120 kVp than at 80 kVp. Since IDR_P_ values were >1 for all fluoroscopic conditions, there was a tendency of both D_P_ and D_S_ to be larger on the top of the phantom than on the lateral side. We confirmed that the percentage dose reduction caused by scatter radiation was larger than that of the primary X-ray beam on the lateral side of the phantom.

The scatter radiation dose did not contribute as much to the radiation dose as did the primary X-ray beam. However, the scatter radiation dose was important to radiation protection for personnel exposure and contributed to patient exposure. The results of the IDR_S/T_ and the IDR_P_ showed that the quantity of scattering was higher on the top of the patient. We defined that the quantity of scattering (scatter to primary ratio) was the integrated dose for a scatter tail section 100 mm in length, which indicates how much the primary X-rays are scattered by the object. The integrated dose of a section 100 mm in length was equal. The quantity of scattering was shown by the IDR_S/T_ and IDR_P_. The IDR_S/T_ reflected the the quantity of scattering. Table 2 shows that the quantity of scattering was higher on the top of the phantom than on the lateral side for each tube voltage. IDR_P_ was defined as the integrated dose ratio between the top and lateral side of the phantom. Table 3 shows that the IDR_P_ values were >1 and the IDR_PS_ values were higher than the IDR_PP_ values, which means that the integrated dose increments of the primary X-ray beam were larger than the scatter tail dose on the top of the phantom for each tube voltage. There was a tendency for the quantity of scattering on the top of the phantom to be higher than that of the lateral side, which was reflected in the results of the IDR_P_ that were similar to the results mentioned above for the IDR_S/T_. In operating the biopsy needle, the physician stood on the lateral side of the patient, and the physician’s hands were often in front of the patient. It has been previously shown that the exposure was greater to the physician’s hands than to other parts [4, 5, 31]. This finding was attributed to the fact that not only were the physician’s hands located near the primary X-ray beam but also that the scatter radiation dose was larger on the top than on the lateral side as shown in this study.

The IDR_V_ values were larger on the lateral side of the phantom than on the top, the difference in IDR_V_ values was caused by absorption inside the phantom and the X-ray beam travel length from the tube focal spot to the phantom surface for each measurement position. A previous study [32] reported that peripheral doses increased drastically as the cylindrical acrylic phantom diameter decreased. The results of IDR_V_ were affected using the chest phantom. Furthermore, the fluoroscopic conditions affected D_P_ rather than D_S_ because the IDR_VP_ values were greater than the IDR_VS_ values. In other words, the tube voltage dependence of the scatter radiation was less than that of the primary X-ray beam under the same fluoroscopic dose-rate condition. The tube voltage dependence was less on the top of the phantom than on the lateral side. The fluoroscopic conditions were set to be equal to the dose rate as the reference displayed CTDI in this study. Maintenance of the same dose rate by decreasing the tube voltage requires simultaneously increasing the tube current. To reduce an electron’s kinetic energy, the tube current increased to compensate for the low tube voltage. A previous study [30] reported that the relationship between normalized CTDI (per 100 mAs) and tube voltage was nonlinear. This effect was greater than the increase in the tube voltage dependence of the quantity of scattering when using the higher tube voltage; therefore, IDR_V_ values were >1 for all fluoroscopic conditions. Using a low tube voltage in maintaining the same dose rate, the patient exposure increases by the primary X-ray beam; nevertheless, there is no reason to select a low tube voltage and increase the tube current for patient exposure. As mentioned above, it was beneficial to decrease the dose rate using a low tube voltage which was a low quantity of scattering. If it was equal to the patient exposure by reducing the dose rate using a low tube voltage, it would be possible that the scatter dose was reduced in comparison to using a high tube voltage. However, it was necessary to ensure that the image quality needed for CT fluoroscopic procedures was achieved because the dose rate was also related to image quality [33, 34].

Our results indicated that the scatter radiation dose characteristic had a high dependence on positioning and a low dependence on tube voltage relative to the primary X-ray beam under the same fluoroscopic dose-rate condition. To reduce personnel exposure during CT fluoroscopic procedures, it was necessary to consider the fluoroscopic conditions and physician position for the patient. A lead drape would be useful for scatter radiation dose reduction [7, 35, 36], and a needle placement device has been reported to reduce the exposure to physicians’ hands [36–38]. It should be noted that the scatter radiation dose in front of the patient was larger on the periphery, particularly to the physicians’ hands during CT fluoroscopic procedures.

In this study, we did not adjust the CT fluoroscopic conditions on the basis of patient factors, such as body weight, body part, and supine or prone position. Further investigations are needed to clarify the potential differences in patient surface dose and physician exposure at the clinical site that may result from different patient factors. A limitation of our study was that the energy-dependent response of the OSL material demanded the use of an appropriate energy-dependent correction factor. The effective atomic number of the OSL material causes an over-response at lower tube voltage settings, so the energy-dependent correction factor was applied to OSL results for tube voltage [16–19]. Because the absolute values of the percent differences between the readings of the OSL dosimeter and ionization chamber were higher for the top and lateral areas at 120 kVp relative to those at 80 kVp, we believe that the energy dependence of OSL was affected. The energy dependence of photon detection efficiency should be carefully considered when using the dosimeter without the ionization chamber. Tables 2 and 3 show that the percentage of the integrated dose for the scatter tail section was larger than that of the primary X-ray beam at 120 kVp. For CTDIs that were equal at 120 kVp and 80 kVp as displayed on the CT console, the CT fluoroscopic X-ray tube current at 120 kVp was considerably lower than that at 80 kVp. The scatter tail dose was caused by scatter radiation that had lower effective energy than that of the primary X-ray beam. Consequently, correction of the energy dependence was incomplete for measurement of the integrated dose of the scatter tail section. Table 1 shows that the OSL dosimetry reading was smaller for the ionization chamber at 120 kVp than at 80 kVp, which meant that the scatter tail dose was insufficient for the energy dependence correction and was large relative to that of the primary X-ray beam dose. We intend to investigate the influence of the energy dependence of the OSL dosimeter under low-dose conditions in the future. The energy of the photon incident on the OSL dosimeter differs depending on the measurement position and X-ray beam filter associated with the CFOV, and calibration measurements may be performed properly using an ionization chamber for each measurement condition.

In conclusion, we showed that it was possible to evaluate scatter radiation during CT fluoroscopy through measurement of the X-ray dose profiles determined using OSL dosimeters. The evaluation of IDRs suggested that the scatter radiation dose had a high dependence on positioning and a low dependence on tube voltage relative to the primary X-ray beam under the same fluoroscopic dose-rate condition. The results provided evidence supporting the necessity of radiation protection methods used during CT fluoroscopy in previous studies. Reduction of scatter radiation was achieved using fluoroscopic conditions giving a low dose rate similar to that needed to reduce exposure to patients. The use of a low tube voltage is recommended under fluoroscopic conditions because the quantity of scattering was lower in this study. A lead drape will reduce scatter radiation of the primary X-ray beam from a patient. Needle placement is a valid method for reducing the exposure to a physician’s hands when they are held in front of a patient. Physicians who perform CT fluoroscopic procedures should consider using these methods to reduce radiation exposure to themselves during examinations.
